# Gender Differences in Admissions and In-Hospital Outcomes of Patients With Acute Coronary Syndromes During the Coronavirus Disease 2019 Pandemic

**DOI:** 10.7759/cureus.23286

**Published:** 2022-03-18

**Authors:** Leonard Simoni, Ilir Alimehmeti, Astrit Ceka, Mirald Gina, Ermir Tafaj, Alban Dibra, Artan Goda

**Affiliations:** 1 Cardiovascular Disease, University Hospital Center Mother Teresa, Tirana, ALB; 2 Occupational Health, Faculty of Medicine, University of Medicine, Tirana, ALB

**Keywords:** covid-19 pandemic, in-hospital outcomes, hospitalizations, gender differences, acute coronary syndromes

## Abstract

Background

The incidence of acute coronary syndromes (ACS) decreased during the coronavirus disease 2019 (COVID-19) pandemic. Few studies have investigated gender differences in ACS admissions and outcomes during pandemics and have presented divergent results. This study aimed to investigate the effect of the COVID-19 pandemic on male and female hospitalizations and in-hospital outcomes in patients presenting with ACS.

Methodology

We designed a retrograde, single-center trial gathering data for ACS hospitalizations during the lockdown (March 9, 2020, to April 30, 2020) compared with the same timeframe of 2019. ACS hospitalizations were subgrouped as ST-elevation myocardial infarction (STEMI), non-STEMI (NSTEMI), and unstable angina (UA). We calculated the incidence rate ratio (IRR) to compare all-ACS and subgroups for male and female hospitalizations and the risk ratio (RR) to compare overall male/female mortality.

Results

This study included 321 ACS patients (238 males, 83 females) during the COVID-19 lockdown and 550 patients (400 males, 150 females) during 2019. The IRRs of all-ACS/males/females were significantly lower during the COVID-19 period at 0.58 (95% confidence interval (CI) = 0.44-0.76), 0.59 (95% CI = 0.43-0.75), and 0.55 (95% CI = 0.37-0.74), respectively. The IRR for STEMI was significantly lower among females (0.59 (95% CI = 0.39-0.89)), but not among males (0.76 (95% CI = 0.55-1.08)) The IRR for NSTEMI was not significantly lower, meanwhile it was significantly lower for UA among both males and females. The overall ACS mortality increased during the COVID-19 period (7.4% vs. 3.4%; RR = 2.16 (95% CI = 1.20-3.89)). Important increase was found in males (7.45% vs. 2.5%; RR = 3.02 (95% CI = 1.42-6.44)), but not in females (7.2% vs. 6%; RR = 1.20 (95% CI = 0.44-3.27).

Conclusions

The admissions of ACS reduced similarly in males and females during the COVID-19 pandemic. The admissions of STEMI reduced predominantly in females. We identified a substantial increase in the overall ACS mortality, but predominantly in males, reducing the differences between males and females. Further studies are necessary to better understand the increase in male mortality during the pandemic.

## Introduction

Studies from all over the world have documented an important reduction in admissions and related invasive procedures in patients with acute coronary syndromes (ACS) during the first wave of the coronavirus disease 2019 (COVID-19) pandemic [1-8]. During this period, worldwide, highly restrictive measures (physical distancing and periods of total lockdown) were adopted to prevent the spread of the virus.

In Albania, during the total lockdown (March 9, 2020, first COVID-19 case [9], until April 30, 2020 [10]), considerable reductions in ACS admissions (-41.6%), coronary angiography (-42.5%), percutaneous coronary interventions (-42.3%) associated with a 2.4-fold increase in in-hospital deaths (3% vs. 7.4 p = 0.024) and a 2.6-fold increase in the incidence of cardiogenic shock (13.1% vs.5.1%; p < 0.0001) compared with the same period of 2019 were reported [11]. In another study treating only ST-elevation myocardial infarction (STEMI), a significant drop in hospitalizations (-28%), representing incidence rate ratio (IRR) of 0.719 (p = 0.033), and revascularization procedures in Albania was observed [12]. Meanwhile, a substantial increase in the STEMI mortality rate (risk ratio (RR) = 1.91; p = 0.037) and cardiogenic shock (RR = 1.70; p = 0.025) during the pandemic outbreak was identified [12].

To date, studies regarding the influence of gender on ACS admissions and treatment document that females, especially those diagnosed with acute myocardial infarction (AMI), present later in hospital from symptom onset [13,14] due to various factors, including social and psychological factors [15,16].

Few studies have been conducted to investigate the gender differences in patients hospitalized with ACS during the COVID-19 pandemic with divergent results regarding the admission frequency, invasive procedures, and outcomes [3,17-19]. Therefore, we undertook this study to investigate the gender differences in hospitalizations and in-hospital outcomes of patients with ACS during the COVID-19 pandemic.

## Materials and methods

We designed a retrospective, single-center trial by gathering data for ACS hospitalizations during the lockdown (March 9, 2020, to April 30, 2020) compared with the same timeframe of 2019. ACS hospitalizations were subgrouped as STEMI, non-STEMI (NSTEMI), and unstable angina (UA). STEMI was diagnosed using the Fourth Universal Definition of Myocardial Infarction criteria [20]. Non-ST-elevation ACS were diagnosed according to the European Society of Cardiology (ESC) 2019 guidelines [21]. The study complies with the Declaration of Helsinki. Informed consent was waived because of the retrospective nature of the study.

Basal characteristic data including age and gender; cardiovascular risk factors including hypertension, type 2 diabetes mellitus, dyslipidemia, and smoking; comorbidities including previous coronary artery disease (CAD) (prior myocardial infarction, percutaneous or surgical coronary revascularization), dilated cardiomyopathy, stroke, and impaired renal function were collected. All coronary angiographic data including the stenotic vessels (stenosis over 50% of diameter), the number of stenotic vessels, vessels with no significant stenosis, and all revascularization procedures including percutaneous coronary intervention (PCI) and coronary artery bypass surgery (CABG) were collected and analyzed.

Admission and in-hospital outcomes

The primary outcomes were overall and weekly IRR of all-ACS and subgroups (STEMI, NSTEMI, UA) admissions and related invasive procedures, in-hospital all-cause mortality, and cardiogenic shock (CSH), respectively, for males and females between the COVID-19 lockdown and control periods. Secondary outcomes were differences between periods for each gender including cardiac troponin I (cTnI) on admission (normal values = 0.00-1.00 ng/mL), time from symptom onset to intensive care unit (ICU) admission, time from ICU admission to sheath insertion, and left ventricular ejection fraction (at discharge).

We calculated the percentage of changes in the overall rate of admissions for ACS and related procedures, as well as the percentage changes in weekly ACS admission comparing the lockdown and control periods. The weekly percentage changes were calculated for seven weeks (March 9 to April 26, 2020) for both periods.

The rate ratio for different genders of all-ACS and subgroups (STEMI, NSTEMI, UA) admissions/procedures between the lockdown and the control period are shown as incidence rate ratios (IRR) which were calculated by comparing the incidence ratios of all-ACS and subgroups (STEMI, NSTEMI, UA) admissions or procedure/day during both periods. IRR is presented as 95% confidence intervals (95% CIs). The ratios of different genders for all-ACS deaths and CSH rate (in percentage) between the study and control periods are shown as RRs with 95% CIs.

Statistical analyses

Continuous variables regarding demographic characteristics, procedural, and hospital outcomes are presented as mean ± standard deviation (SD) and compared using t-test. Categorical variables are represented as percentage and RR with 95% CI and compared using the chi-square (χ^2^) test. Poisson regression (STEMI admissions per week model) was used to calculate the IRR for male and female admissions and procedures between the lockdown and control periods. IRR between the groups is presented as 95% CI. A two-sided p-value of <0.05 was considered statistically significant. The statistical analysis was performed using SPSS version 21 (IBM Corp., Armonk, NY, USA).

## Results

Patients characteristics

Overall, 871 patients admitted with the diagnosis of ACS were included in this analysis. Of them, 638 were males (73.3%). In total, 238 were hospitalized during the lockdown and 400 during the control period; and of the 233 (26.7%) females, 83 were hospitalized during the lockdown and 150 during the control period. General characteristics are shown in Table 1. There were no differences between patients hospitalized during the lockdown and control periods both in males and in females regarding age and cardiovascular risk factors.

**Table 1:** Baseline demographic, clinical, and procedural characteristics of the study population according to gender groups. *To determine statistical significance continuous variables were presented as mean ± SD and compared using t-tests; meanwhile, categorical variables were presented as percentages compared using the chi-square test. STEMI: ST‐segment elevation myocardial infarction; CAD: coronary artery disease; PCI: percutaneous coronary intervention; CABG: coronary artery bypass grafting; CMP: cardiomyopathy; LM: left main; LAD: left anterior descending; LCx: left circumflex; RCA: right coronary artery

Variables	Study period, Male 238 patients n (%)	Control period, Male 400 patients n(%)	P-value*	Study period, Female 83 patients n (%)	Control period, Female 150 patients n (%)	P-value*
Age, years (SD)	64.54 (10.39)	64.82 (10.64)	0.864	67.80(10.52)	69.15 (10.15)	0.338
STEMI	121 (50.8%)	158 (39.5%)	0.007	35 (42.2%)	59 (39.3%)	0.777
NSTEMI	49 (20.6%)	70 (17.5%)	0.388	15 (18.1%)	25 (16.7%)	0.927
UA	68 (28.6%)	172 (43%)	<0.001	33 (39.7%)	66 (44%)	0.625
Diabetes mellitus	100 (42.1%)	163 (40.8%)	0.817	36(43.4%)	82 (54.7%)	0.130
Hypertension	213 (89.5%)	363 (90.8%)	0.705	79 (95.2%)	139 (92.7%)	0.638
Dyslipidema	132 (55.5%)	208 (52%)	0.444	47 (56.6%)	82 (54.7%)	0.880
Smoking	89 (37.4%)	159 (39.8%)	0.613	3 (3.6%)	14 (9.3%)	0.179
Previous CAD	44 (18.5%)	99 (24.8%)	0.082	9 (10.8%)	34 (22.7%)	0.032
Dilated CMP	8 (3.8%)	35 (8.8%)	0.014	4 (4.8%)	10 (6.7%)	0.779
Impaired renal function	37 (15.6%)	49 (12.3%)	0.289	6 (7.2%)	22 (14.7%)	0.143
Previous stroke	9 (3.8%)	17 (4.3%)	0.934	2 (2.4%)	9 (6.0%)	0.360
Coronary angiography	214 (89.9%)	369 (92.3%)	0.384	69 (83.1%)	127 (84.7%)	0.904
Refused coronary-angiography	17 (7.1%)	21 (5.3%)	0.421	13 (15.7%)	17 (11.3%)	0.453
CAD (n % angiography.)	202 (94.4%)	331 (90.0%)	0. 072	53 (76.8%)	106 (83.5%)	0.344
One-vessel CAD (n % angiography)	54 (25.2%)	85 (23%)	0.617	18 (26.1%)	34(26.7%)	0.945
Two-vessel CAD (n % angiography)	49 (22.9%)	123 (33.3%)	0.010	19 (27.5%)	30 (23.6%)	0.665
Three-vessel CAD (n % angiography)	99 (46.3%)	123 (33.3%)	0.003	16 (23.2%)	42 (33.1%)	0.199
LM disease (n % angiography)	31 (14.5%)	36 (9%)	0. 141	6 (8.7%)	6 (4.7%)	0.448
No critic stenoses (n % angiography)	12 (5.6%)	38 (9.8%)	0.127	16(23.2%)	21(16.5%)	0.385
PCI (% patients)	135 (63.1%)	223 (60.4%)	0.585	40 (58.0%)	80 (63.0%)	0.592
PCI of LAD (% of PCI)	78 (57.8%)	128 (57.4%)	0.944	27 (67.5%)	55 (68.5%)	0.845
PCI of LCX (% of PCI)	37 (27.4%)	62 (27.8%)	0.941	10 (25.0%)	21 (26.3%)	0.890
PCI of RCA (% of PCI)	57 (42.2%)	101 (45.3%)	0.674	12 (30%)	34 (42.5%)	0.231
CABG recommended (% patients)	51(21.2%)	81 (20.3%)	0.673	9 (13%)	16 (12.6%)	0.930
CABG performed (% patients)	20 (9.3%)	24 (6%)	0.276	3 (3.3%)	6 (4.7%)	0.904
CAD with medical treatment (n % patients)	17 (7.9%)	36 (9.8%)	0.559	8 (11.6%)	16 (12.6%)	0.838

Gender differences in hospitalizations for ACS during the COVID-19 lockdown and control periods

**Figure 1:**
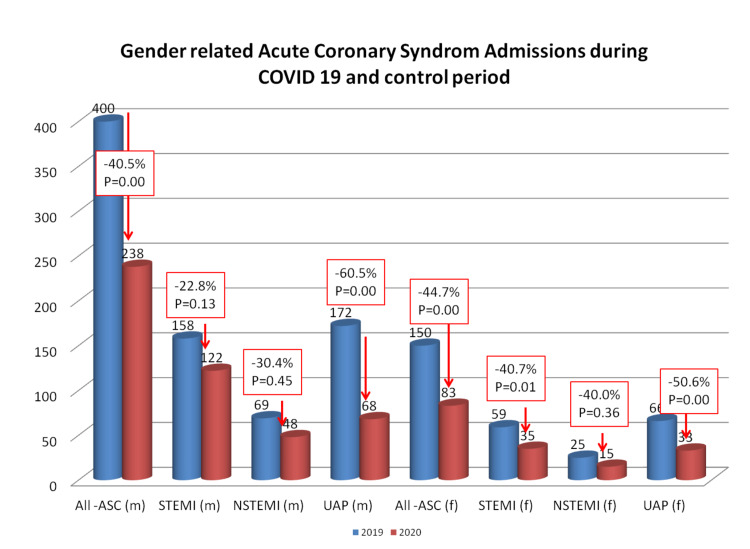
Gender-related coronary syndrome admissions during the COVID-19 and control periods. Male and female all-ACS, STEMI, non‐STEMI, and UA admissions during the COVID-19 period (red bars) and control period (blue bar) showing the percentage reduction and statistical significance. ACS: acute coronary syndrome; STEMI: ST‐segment elevation myocardial infarction; UA: unstable angina; COVID-19: coronavirus disease 2019

**Figure 2:**
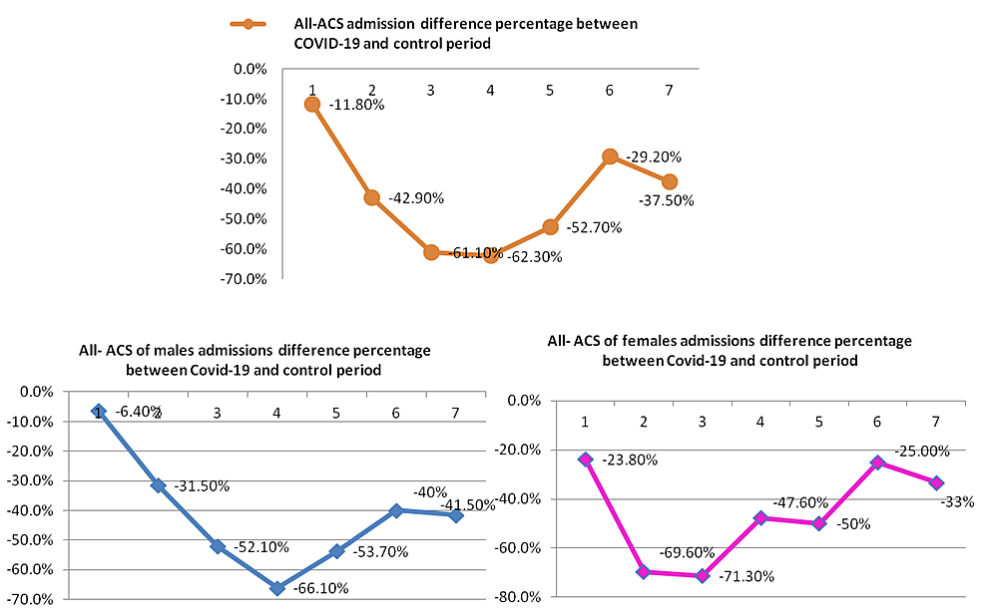
All/male/female ACS admissions difference percentage between the COVID-19 and control periods. The weekly admission difference percentage between the lockdown and control periods was obtained for all-ACS, showing an important reduction in all/male/female admissions during the second to the fifth week of the study compared to the control period. ACS: acute coronary syndromes; COVID-19: coronavirus disease 2019

The reduction in overall ACS was observed to be 40.5% in males and 44.7% in females. (Figure 1) The weekly IRR in hospitalizations for ACS during the lockdown and study periods both in males and females was statistically significant (0.60, 95% CI = 0.43-0.75, p < 0.001; and 0.55 (95% CI = 0.37-0.77, p = 0.001, respectively) (Figure 2 and Table 2). Admissions for males and females according to the ACS type, coronary angiography, and PCI during the lockdown and control period are shown in Table 1 and Figure 1. The IRR for STEMI was significantly lower among females (0.59 (95% CI = 0.39-0.89)), but not in males (0.76 (95% CI = 0.55-1.08)). The IRR for NSTEMI was not significantly lower for both males and females (0.68 (95% CI = 0.41-1.08; and 0.70 (95% CI = 0.36-1.19), respectively), meanwhile, for UA, it was significantly lower for both males and females. The greatest fall in all-ACS admissions was observed during the third week for females (-71.3%) and the fourth week for males (-66.1%) during the pandemic outbreak (Figure 2).

**Table 2:** The incidence rate ratio of male/female admissions and invasive procedures. ^†^IRR for admissions and invasive procedures obtained from the analyses of seven weeks during the lockdown and control periods presented as IRR and 95% CI. STEMI: ST‐segment elevation myocardial infarction; PCI: percutaneous coronary intervention; IRR: incidence rate ratio; CI: confidence interval *To determine statistical significance for the comparison regarding male/female ACS and subgroups admissions and procedures, the Poisson regression (admissions/procedure per week model) was used.

Admission presentation and procedures	COVID‐19	Control	IRR IRR (95% CI)†	P‐value*
ACS males (% all patients)	238 (74%)	400 (73%)	0.60 (0.43-0.75)	<0.001
ACS females (% all patients)	83 ( 26%)	150 (27%)	0.55 (0.37-0.77)	0.001
STEMI males (% all males)	121 (50.8%)	158 (39.5%)	0.77 (0.55-1.07)	0.131
STEMI females (% all females)	35 (42.2%)	59 (39.3%)	0.59 (0.39-0.89)	0.013
NSTEMI males (% all males)	49 (20.6%)	70 (17.5%)	0.70 (0.36-1.19)	0.452
NSTEMI females (% all females)	15 (18.1%)	25 (16.7%)	0.60 (0.27-1.10)	0.360
UA males (% all males)	68 (28.6%)	172 (43%)	0.39 (0.23-0.51)	<0.001
UA females (% all females)	33 (39.7%)	66 (44%)	0.55 (0.33-0.84)	0.001
Angiography males	214 (89.9%)	369 (92.3%)	0.58 (0.43-0.76)	0.009
Angiography females	69 (83.1%)	127 (84.7%)	0.54 (0.38-0.74)	0.007
PCI males	135 (63.1%)	223 (60.4%)	0.61 (0.39-0.90)	0.031
PCI females	40 (58.0%)	80 (63.0%)	0.50 (0.31-0.78)	0.002

The proportion of male STEMI hospitalizations was considerably higher during lockdown compared to the control period (50.8% vs. 39.5%; p = 0.001), meanwhile, hospitalizations for UA reduced (28.6% vs. 43%; p < 0.001; Table 1). There were no differences in the proportion of female admissions with STEMI, NSTEMI, and UA between the study and control periods.

Coronary intervention findings

The number of male and female patients undergoing coronary angiography during the lockdown compared with the control period significantly dropped by 42% and 46%, respectively, with a weekly IRR of 0.58 (95% CI = 0.43-0.76; p = 0.009) for males and 0.54 (95% CI = 0.38-0.74, p = 0.007) for females. The number of male and female patients undergoing PCI also dropped by 39% and 50%, respectively, during the lockdown, representing an IRR of 0.61 (95% CI = 0.39-0.90; p = 0.031) for males and 0.50 (95% CI = 0.31-0.78; p = 0.002) for females (Table 2).

There were no significant differences in the percentage of male and female patients undergoing coronary angiography, PCI, and CABG between the two periods. Even though for the recommended CABG, a greater proportion of male patients compared to females was observed in both periods at 21.2% versus 13%, respectively, during the COVID-19 period and 20.3% versus 12.6%, respectively, during 2019 (Table 1).

The frequency of three-vessel disease (46.3% versus 33.3%; p = 0.003) was greater among male patients during the lockdown period. No differences were observed regarding the frequency of vessel disease in female patients (Table 1).

In-hospital outcomes

The symptom onset to ICU admission duration was significantly higher during the lockdown than the control period both in males (14.54 ± 17.94 versus 7.08 ± 5.88 hours; p < 0.001) and females (19.76 ± 21.05 versus 7.79 ± 7.63 hours; p < 0.001). The cTnI was higher on admission (21.01 ± 45.11 versus 9.01 ± 24.65 ng/mL; p < 0.001), and ejection fraction was lower (48.24 ± 11.68 versus 50.68 ± 11.33; p = 0.01) only among male patients hospitalized during the lockdown compared with the control period. Conversely, the ICU presentation to sheath insertion time was shorter for male patients (47.8 ± 24.4 versus 57.8 ± 33.42 hours; p = 0.011).

Compared with the control period there was three times higher in-hospital mortality among male patients hospitalized during the lockdown (2.5% versus 7.6%; p = 0.005), with an RR of 3.02 (95% CI = 1.42-6.44; p = 0.032), as well as three times higher incidence of CSH (4.0% versus 12.2%; p < 0.0001). Meanwhile, there was no significant increase of female mortality (7.2% versus 6.0%; p = 0.783) and CSH rate (15.7% versus 8.0%; p = 0.07) between the COVID-19 and control period with RR of 1.20 (95% CI = 0.44-3.27) for mortality and 1.96 (95% CI = 0.94-4.09) for CSH, respectively (Tables 3, 4).

**Table 3:** Male and female in-hospital outcomes between the study and control periods. *To determine statistical significance for the comparison regarding each in-hospital outcome variable were summarized using mean ± SD for continuous variables compared using t-test and frequency and percentage for categorical variables compared using the chi-square test. ACS: acute coronary syndrome; ICU: intensive care unit; cTn I: cardiac Troponin I

Variables	Study period, Male 238 patients n (%)	Control period, Male 400 patients n (%)	P-value*	Study period, Female 83 patients n (%)	Control period, Female 150 patients n (%)	P value*
Symptom onset-ICU time (SD) (hours)	14.54 (17.94)	7.08 (5.88)	<0.001	19.76 (21.05)	7.79 (7.63)	<0.001
ICU-sheath time (SD) (minutes)	47.8 (24.4)	57.8 (33.42)	0.011	54.7 (42.5)	55.0 (33.2)	0.964
cTn I (SD)	21.01 (45.11)	9.01 (24.65)	<0.001	18.64 (38.53)	15.45 (52.58)	0.653
Ejection fraction (SD)	48.24 (11.68)	50.68 (11.33)	0.010	49.01 (13.40)	51.65 (12.40)	0.132
Length of stay (SD)	4.29 (2.49)	6.52 (3.40)	<0.001	4.95 (3.65)	6.94 (3.70)	<0.001
Death	18 (7.6%)	10 (2.5%)	0.005	6 (7.2%)	9 (6.0%)	0.783
Cardiogenic shock	29 (12.2%)	16 (4.0%)	<0.001	13 (15.7%)	12 (8.0%)	0.112

**Table 4:** Overall/male/female major complications risk ratios. ^†^RR for death and CSH was obtained from the comparison of event rate (death/CSH) between the lockdown and control periods and expressed as RR and 95% CI. CSH: cardiogenic shock; RR: risk ratio; CI: confidence interval

Complications	COVID‐19	Control	RR (95% CI)†	P‐value
Death n (% all patients)	24 (%)	19 (%)	2.16 (1.20-3.89)	0.001
Death males	18 (7.6%)	10 (2.5%)	3.02 (1.42-6.44)	0.004
Death females	6 (7.2%)	9 (6.0%)	1.20 (0.44-3.27)	0.714
CSH n (% all patients)	42 (21.2%)	28 (12.4%)	2.57 (1.63-4.06)	<0.001
CSH males	29	16	3.05 (1.69-5.49)	<0.001
CSH females	13	12	1.96 (0.94-4.09)	0.074

## Discussion

In Albania, previous studies have documented that the admissions for ACS, subgroups, and related invasive procedures declined significantly during COVID-19 compared to the control period (2019), associated with a 2.4-fold increase in in-hospital deaths [11,12]. In this study, reduction in ACS was observed in both males and females (-40.5%, p = 0.002, and -44.7%, p = 0.001, respectively). A significant reduction was observed in UA among both males and females (-60.5% and -50.6%, respectively), and only in female STEMI admissions (-40.7%). The greatest decline in hospitalizations for males and females was observed during the second to the fifth week following the pandemic outbreak. Moreover, important reductions were observed in males and females in coronary angiography and PCI procedures. Similar proportions of males and females undergoing coronary angiography and PCI during both periods were observed because there were no changes in ACS/STEMI revascularization protocols during the COVID-19 pandemic lockdown in our center. A significant increase in in-hospital mortality and CSH in male patients admitted during the COVID-19 period was observed.

Our results are consistent with studies reporting a reduction in ACS admissions among males as well as in females. Barbero et al. found a significant reduction in overall ACS admissions in 15 hospitals in Northern Italy in an inter-year analysis by 42% for females and 24% for males [17].

In our view, the possible explanations of the reduction among both males and females are the fear of catching the virus in medical establishments, the call of self-isolation, and physical distance from the authorities leading to refusing or not seeking medical care. Another possible explanation is the reduction of physical activities during the lockdown period, reducing the possible triggers for myocardial ischemia, followed by a reduction in ACS admissions.

The greatest reduction in both males and females was observed in UA admissions. A change in the profile of admitted patients was observed, mainly among male patients. The proportion of male hospitalizations with STEMI was significantly higher during lockdown (50.8% versus 39.5%; p = 0.001), whereas admissions for UA were lower (28.6% versus 43%; p < 0.0001) compared to 2019 (Table 1). This can be explained by the fact that those who presented at the hospital to receive medical care were in more severe conditions and presented with more symptoms during the pandemic.

Congruent to other studies, we documented an important reduction in female STEMI admissions with an IRR of 0.59 (95% CI = 0.39-0.89), but not for male patients with an IRR of 0.76 (95% CI = 0.55-1.08). Huynh et al. in Sweden reported that the incidence of acute myocardial infarction (AMI) decreased mostly in females aged over 70 years with an IRR of 0.56 (95% CI = 0.40-0.78) during the COVID-19 pandemic compared to the reference period (2017-2019) than among males (0.97, 95% CI = 0.77-1.23) [18]. De Rosa et al. [3] in Italy found a higher reduction for females admitted with STEMI (41.2%; p = 0.011) than males (17.8%; p = 0.191). Further, Pacheco et al. [19] documented a greater reduction in females compared to males of myocardial infarction admissions (level effect = 0.595 (0.566-0.627) versus 0.532 (0.502-0.564)).

On the other hand, other studies have presented divergent results. Mesnier et al. [6] found no differences between genders in the reduction (-30%) of the admissions for AMI compared to the control period, presenting an IRR of 0.70 (95% CI = 0.61-0.80) for men and 0.70 (0.56-0.88) for females. Moreover, no differences were observed by Solomon et al. [22] in Northern California between genders in the reduction of AMI admissions compared to the control period. Meanwhile, Moreno et al. in a study conducted in Madrid, Spain, found a significant reduction in males (-28.7%), but not in females during the pandemic compared with the pre-pandemic period [23].

The reduction in the admissions of females with STEMI in our study could be related to greater refutation and hesitation in seeking medical care during the COVID-19 pandemic. In our study, we observed a significant increase in the symptom onset to ICU admission in both genders compared to the control period, but there was a trend to a higher increase in female delay compared to males during the COVID-19 pandemic (19.76 ± 21.05 versus 14.54 ± 17.94 hours; p = 0.150). Previous studies have suggested that males and females show different behavior during AMI [15,16]. Females with STEMI are associated with significantly larger delays in seeking medical care related to psychosocial factors such as not disturbing, being a problem to others, and older age [13,14]. These factors probably worsened during the pandemic, especially due to the recommendations of self-isolation and physical distancing, impacting the admissions and the delay in hospital presentation and treatment.

In our study, during the “normal” 2019 year, compared to males, females subjected to the so-called gender paradox of coronary disease tended to present later (424.72 ± 352.6 versus 467.46 ± 457.63), had worse outcomes, had higher mortality (6.0% versus 2.5%; p = 0.01), but tended to have smaller coronary disease extent (more than one-vessel CAD, less than two and three-vessel CAD) and better ejection fraction (50.68 ± 11.33 vs. 51.65 ± 12.40) (Table 5). The paradox of coronary disease is described in other studies conducted worldwide [24] and previously in Albania [25].

**Table 5:** Study and control in-hospital outcomes between males and females. *To determine statistical significance variables were summarized using mean ± SD for continuous variables compared using t-tests, and percentages for categorical variables were compared using chi-square tests. ACS: acute coronary syndrome; ICU: intensive care unit; cTn I: cardiac Troponin I; SD: standard deviation

Variables	Study period, Male 238 patients n (%)	Study period, Female 83 patients n (%)	P-value*	Control period, Male 400 patients n (%)	Control period, Female 150 patients n (%)	P-value*
Symptom onset-ICU time (SD), hours	14.54 (17.94)	19.76 (21.05)	0.08	7.08 (5.88)	7.79 (7.63)	0.673
cTn I (SD)	21.01 (45.11)	18.64 (38.53)	0.235	9.01 (24.65)	15.45 (52.58)	0.452
Ejection fraction (SD)	48.24 (11.68)	49.01 (13.40)	0.620	50.68 (11.33)	51.65 (12.40)	0.383
Death	18 (7.6%)	6 (7.2%)	0.891	10 (2.5%)	9 (6.0%)	0.015
Cardiogenic shock	29 (12.2%)	13 (15.7%)	0.672	16 (4.0%)	12 (8.0%)	0.112

The gender paradox of coronary disease was not observed during the COVID-19 period because of similar outcomes between males and females, CSH (12.2% versus 15.7%; p = 0.67), and mortality (7.6% versus 7.2%; p = 0.89) (Table 5), essentially because of the increase of CSH (12.2 versus 4%; p = 0.0001) and mortality (7.6% versus 2.5%; p = 0.005) in male patients during the pandemic compared to 2019. Meanwhile, the increase in female mortality was insignificant between the two periods (7.2% versus 6%; p = 0.26), as presented in Table 3.

As mentioned above, such an increase in male mortality leads to a reduction in differences between genders and losing “superiority” toward female patients regarding the ACS outcomes during the COVID-19 period. In our view, increased male mortality is related to a worse patient profile during the COVID-19 period as it is documented in previous studies for the overall ACS [11] and STEMI [12] mortality in Albania. The main male presentation during the study compared to the control period was STEMI (50.8% versus 39.5%; p = 0.007). The female presentation did not differ between periods. Male patients had a greater myocardial injury, as shown by the higher level of cTnI at presentation (21.01 ± 45.11 versus 9.01 ± 24.65; p = 0.0001) and lower LVEF (48.24 ± 11.68 versus 50.68 ± 11.33; p = 0.010) during the COVID-19 period. On the other hand, the level of cTnI and the LVEF were similar in female patients during both periods.

In our study, we found a reduction in ACS admissions and related invasive procedures in both males and females during the COVID-19 pandemic, mainly because of the reduction of UA (less severe) presentation in both genders. We found a reduction in female STEMI presentations, probably related to their specific psychological and social factors combined with pandemic factors. We documented a significant increase in male mortality, probably related to a greater proportion of STEMI patients, and greater myocardial damage (high cTnI and lower LVEF). The collateral damage of the pandemic, ACS not presented at all, or presented late, is extended in both genders, impacting future cardiovascular morbidity and mortality.

Strengths and limitations

The study included all consecutive patients, males and females, admitted with ACS in our center in two different periods, namely, pre-pandemic and pandemic (lockdown) periods, presenting the entire view of different gender admissions, management, invasive treatment, and complications. However, our study is a retrospective study and all data were taken from medical files with possible known biases [26].

As a reference center and the largest tertiary public center with primary PCI service, all ACS patients throughout Albania (regional hospitals) are normally transferred to our center. It is not clear the proportion of patients not transferred in Tirana and those treated in regional hospitals.

On the other hand, the patients diagnosed with COVID-19 and ACS were treated in another hospital and were not included in our study. To our knowledge, the number of patients with COVID-19 and ACS was small during the lockdown period, but the impact on admissions and in-hospital outcomes remains unknown.

## Conclusions

Admissions for ACS were reduced similarly in males and females during the COVID-19 pandemic. Main reductions were observed in the UA group in both genders and STEMI predominantly in females, probably related to their specific psychological and social combined with pandemic factors. We identified a substantial increase in the overall ACS male mortality reducing the differences between genders. Further studies are necessary to better understand the increase in male mortality during this period.
